# Focus on Baseline LDL-C and Patient Risk, Not Drug Type: A Perspective on Alirocumab vs Evolocumab

**DOI:** 10.31083/RCM50430

**Published:** 2026-04-21

**Authors:** Pierre Sabouret, Domenico Mario Giamundo, Mamas Mamas

**Affiliations:** ^1^Department of Cardiology, Sorbonne University, 75013 Paris, France; ^2^Department of Systems Medicine, “Tor Vergata” University, 00133 Rome, Italy; ^3^Department of Cardiology, Royal Stoke University Hospital, University Hospitals of North Midlands NHS Trust, ST4 6QG Stoke-on-Trent, UK; ^4^Keele Cardiovascular Research Group, Centre for Prognosis Research, Keele University, ST5 5BG Keele, UK

## 1. Introduction

A recent meta-analysis suggests greater clinical benefit of Proprotein convertase subtilisin/kexin type 9 inhibitors (PCSK9i) among patients with baseline Low-Density-Lipoprotein cholesterol (LDL-C) levels of 100 mg/dL or higher, with cardiovascular mortality reduction observed in the alirocumab-treated post-acute coronary syndrome (ACS) population included in the ODYSSEY OUTCOMES [1]. Importantly, no head-to-head randomized trials have directly compared alirocumab and evolocumab across different baseline LDL-C strata, and available network meta-analyses do not demonstrate meaningful differences in relative risk reduction for major cardiovascular events (MACE) between the two agents. Therefore, any apparent heterogeneity in clinical benefit according to baseline LDL-C should not be interpreted as evidence of intrinsic differences in drug efficacy.

**Fig. 1.**
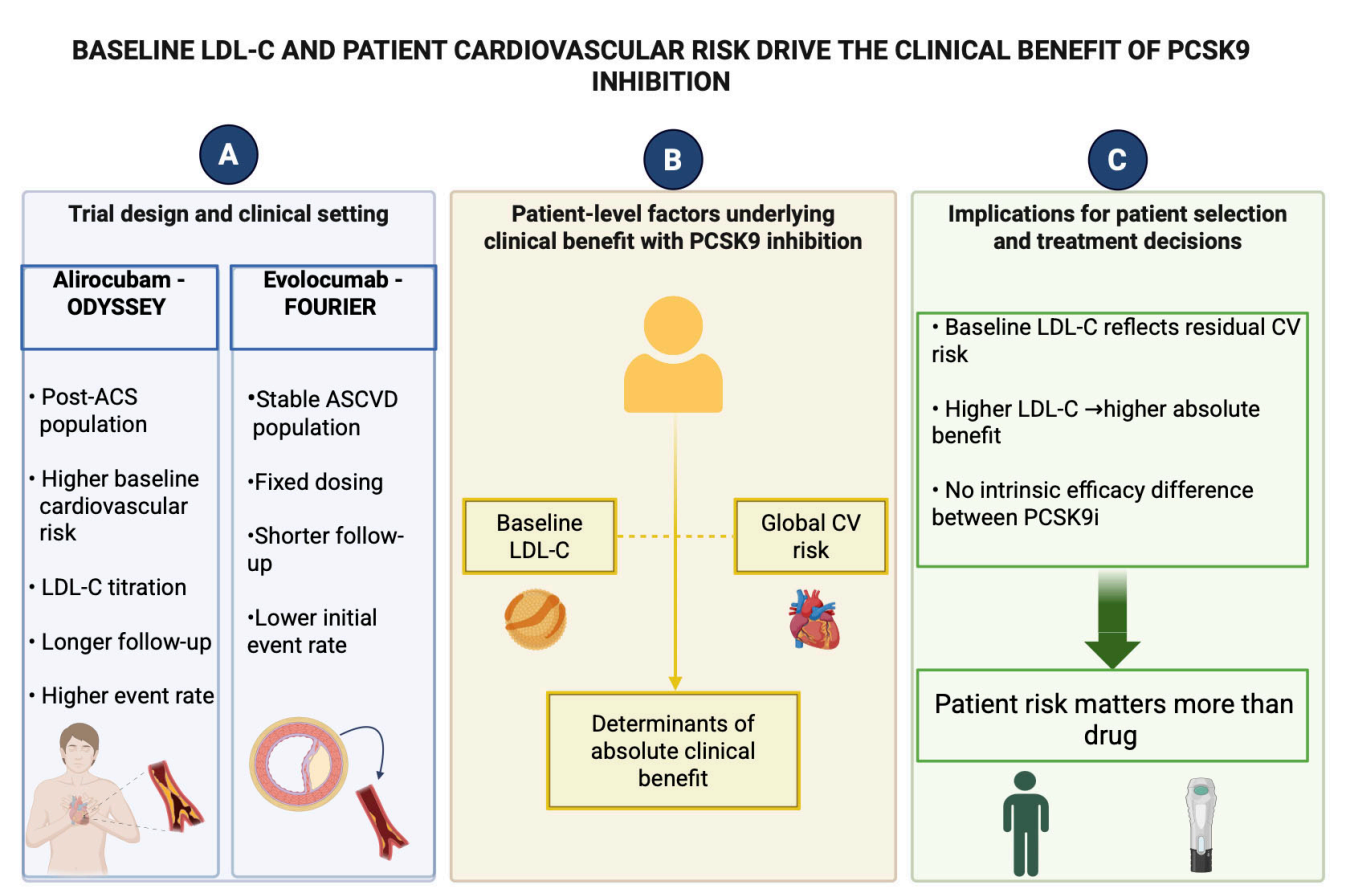
**Baseline LDL-C and patient cardiovascular risk drive the clinical benefit of PCSK9 inhibition**. (A) Summarizes the main differences in trial design and clinical setting between alirocumab in ODYSSEY OUTCOMES and evolocumab in FOURIER. ODYSSEY OUTCOMES enrolled patients after acute coronary syndrome, characterized by higher baseline cardiovascular risk, LDL-C titration and longer follow-up, whereas FOURIER included patients with stable atherosclerotic cardiovascular disease treated with fixed-dose evolocumab and lower initial event rates. Figure legends: ACS (acute coronary syndrome), LDL-C (Low-Density-Lipoprotein cholesterol), ASCVD (stable atherosclerotic cardiovascular disease), CV (cardiovascular), PCSK9i (Proprotein convertase subtilisin/kexin type 9 inhibitors). (B) Schematically illustrates the patient-related factors underlying the magnitude of absolute clinical benefit with PCSK9 inhibition. Baseline LDL-C levels and global cardiovascular risk are shown as coexisting and complementary determinants that define residual cardiovascular risk and, consequently, the potential absolute benefit of treatment. The schematic emphasizes that clinical benefit is primarily driven by patient characteristics rather than by differences between PCSK9 inhibitor molecules. (C) Outlines the clinical implications, indicating that baseline LDL-C reflects residual cardiovascular risk and potential absolute benefit, without intrinsic efficacy differences between PCSK9 inhibitors; therefore, patient risk profile rather than molecule choice should guide clinical decision-making.

This Editorial does not aim to compare the intrinsic efficacy of alirocumab and evolocumab, but rather to discuss how baseline LDL-C, residual patient risk, trial design, and clinical context modulate the observed magnitude of clinical benefit and may contribute to the perception of differential effects between these two PCSK9 inhibitors (Fig. 1).

Both alirocumab and evolocumab provide large and consistent relative risk reductions in MACE across a wide range of baseline LDL-C levels. However, the apparent modulation of absolute benefit by baseline LDL-C is more clearly illustrated in the ODYSSEY OUTCOMES, conducted in a post-ACS setting, than with evolocumab in stable atherosclerotic cardiovascular disease (ASCVD), since the baseline risk was higher and the follow-up longer in ODYSSEY OUTCOMES. These factors directly influence MACE rate, as demonstrated by the randomised controlled trial (RCT) meta-analysis and by comparison between the FOURIER and OLE FOURIER trials [2]. Indirect and real-world comparisons do not support a major intrinsic difference between the two monoclonal PCSK9i; observed gradients by baseline LDL-C mostly reflect trial design, background risk, and targeting strategies rather than molecule-specific biology [3].

## 2. Baseline LDL-C in Pivotal Trials

In ODYSSEY OUTCOMES, patients enrolled after recent ACS had a median baseline LDL-C of approximately 92–93 mg/dL, with enrichment of higher-risk individuals, and were treated with a titration-based alirocumab strategy aimed at achieving very low LDL-C levels. In contrast, FOURIER enrolled patients with ASCVD and a similar average baseline LDL-C, who were treated with fixed-dose evolocumab without titration and achieved LDL-C levels of approximately 30 mg/dL early during follow-up [4]. Thus, despite comparable baseline LDL-C levels, the two programs differed substantially in the clinical setting, baseline cardiovascular risk, treatment algorithm, and event rates factors that are expected to critically influence absolute risk reduction and overall clinical benefit.

## 3. Effect of Baseline LDL-C on Outcomes: Alirocumab

In ODYSSEY OUTCOMES, alirocumab was associated with a significant reduction in MACE, with a pronounced gradient of absolute benefit across baseline LDL-C strata. Patients with baseline LDL-C of 100 mg/dL or higher derived the greatest benefit, including a significant reduction in cardiovascular mortality. Importantly, this finding should not be interpreted as evidence of the superiority of alirocumab over evolocumab, but rather as a consequence of higher baseline cardiovascular risk, longer follow-up, and a treat-to-target strategy in a post-ACS population. In contrast, patients with lower baseline LDL-C experienced smaller absolute risk reductions despite similar proportional LDL-C lowering, consistent with observations from other randomized controlled trials [5]. Meta-regression analyses across PCSK9i and statin trials further support a relationship between higher baseline LDL-C and greater mortality benefit, in line with the cholesterol-years concept and cumulative exposure to cardiovascular risk.

## 4. Effect of Baseline LDL-C on Outcomes: Evolocumab

In FOURIER, evolocumab was associated with marked LDL-C reduction and a consistent relative risk reduction in MACE across a broad range of patient subgroups [4]. Analyses according to LDL-C levels achieved early in follow-up demonstrated a monotonic association between lower achieved LDL-C and lower event rates [6]. In contrast to ODYSSEY OUTCOMES, FOURIER did not show a strong qualitative interaction between baseline LDL-C and relative treatment efficacy, with broadly similar relative risk reductions across baseline LDL-C categories and differences mainly reflected in absolute risk reduction. This pattern is consistent with the inclusion of a lower-risk, stable atherosclerotic cardiovascular disease population and a fixed-dose treatment strategy. Accordingly, the absence of a mortality benefit signal in FOURIER should be interpreted in the context of lower baseline cardiovascular risk, a stable disease setting, and a shorter duration of follow-up, rather than as a lack of efficacy of evolocumab. Overall, FOURIER and its open-label extension support the principle that “lower and longer is better”, highlighting the importance of cumulative exposure to LDL-C lowering over time [2].

## 5. Alirocumab Versus Evolocumab: Head-to-Head and Indirect Data

Network meta-analyses suggest that evolocumab may achieve slightly greater percentage LDL-C reductions than standard alirocumab doses at comparable time points [3]. However, indirect comparisons of cardiovascular outcomes do not demonstrate meaningful differences in relative risk reduction for major adverse cardiovascular events between the two agents after accounting for baseline risk and follow-up duration. Real-world studies in secondary prevention report broadly similar LDL-C reductions and on-treatment LDL-C levels with both agents, despite differences in baseline LDL-C and background lipid-lowering therapy, including more frequent ezetimibe use among patients treated with alirocumab [7]. Safety profiles are largely comparable, with no major differences across clinically relevant adverse events. Taken together, these findings support the interpretation that apparent differences in clinical benefits according to baseline LDL-C are driven predominantly by patient risk profiles, clinical setting (post-ACS vs chronic ASCVD), and treatment strategies, rather than by intrinsic differences between the two monoclonal antibodies [8].

## 6. Clinical Implications and Positioning by Baseline LDL-C

In stable ASCVD with LDL-C ≥70 mg/dL, evolocumab offers consistent risk reduction down to LDL-C levels of approximately 20–30 mg/dL [6], and the choice of the agent may reasonably be driven by access, dosing preferences, and prior experience. At lower baseline LDL-C (70–99 mg/dL), both agents deliver similar proportional LDL-C reductions and relative risk reduction (RRR), but the absolute risk reduction (ARR) is modest, strengthening the argument for careful patient selection and integration of other risk markers such as Lp(a), multivessel coronary disease, polyvascular disease, type 2 diabetes, or recurrent ischemic events. Ultimately, the key message is that baseline LDL-C should be viewed less as a discriminator between alirocumab and evolocumab and more as a marker of residual risk that modulates the yield of intensive PCSK9 inhibition on top of optimal lipid-lowering therapy [2]. From a mechanistic standpoint, higher baseline LDL-C likely reflects longer cumulative exposure to atherogenic burden and greater plaque vulnerability, such that intensive LDL-C lowering in high-risk clinical settings translates into larger absolute risk reductions over time. From a practical perspective, the decision to initiate PCSK9 inhibition should prioritize patients with higher baseline LDL-C and elevated overall cardiovascular risk, particularly those with recent ACS, polyvascular disease, or recurrent ischemic events despite optimal lipid-lowering therapy. In such settings, the choice between alirocumab and evolocumab should be guided primarily by pragmatic considerations, including access, reimbursement policies, dosing preferences, and patient adherence, rather than by expectations of differential efficacy. Future head-to-head randomized trials, specifically enrolling patients with high baseline LDL-C and elevated cardiovascular risk, would be valuable to further explore the long-term comparative effectiveness of PCSK9i in different clinical settings.
